# Older adults with osteoarthritis show lower functional performance compared to those with diabetes or hypertension: Evidence from the SHARE dataset

**DOI:** 10.1016/j.ocarto.2026.100781

**Published:** 2026-03-21

**Authors:** Mahdie Rafiei, Niklas Probul, Jan Baumbach, Linda Baumbach

**Affiliations:** a Institute for Computational Systems Biology, University of Hamburg, Hamburg, Germany; b Computational Biomedicine Lab, Department of Mathematics and Computer Science, University of Southern Denmark, Odense, Denmark; c Department of Health Economics and Health Services Research, University Medical Center Hamburg-Eppendorf, Germany; d Chair of Genome Informatics, Center for Bioinformatics, University of Hamburg, Hamburg, Germany

**Keywords:** Functional test, Osteoarthritis, Diabetes, Hypertension, Physical activity

## Abstract

**Objective:**

To compare objective functional performance among individuals with osteoarthritis (OA), diabetes, and hypertension (HT), alone or in combination.

**Methods:**

We used data from Wave 2 of the Survey of Health, Ageing and Retirement in Europe (SHARE). Participants were grouped into seven disease categories based on the presence of OA, diabetes, and HT, alone or in combination, plus a no-disease reference group. Functional performance was assessed using grip strength and a five-chair rise test for adults <75 years, and grip strength and walking speed for adults ≥75 years. Ordinary least squares regression models adjusted for age, sex, BMI, and educational level estimated group differences.

**Results:**

We included data from 15,222 individuals. Our adjusted analyses showed that individuals <75 years from all groups, except those with hypertension only, showed lower grip strength compared to those with no disease. For the chair stand test, differences were only observed for patients with OA and those with HT and diabetes. Among individuals older than 75 years, patients with only OA, only diabetes, OA and diabetes, and those with all three diseases had lower grip strength. No group differences were seen in the walking speed test. The estimated differences of the patient groups were small and close, often with overlapping confidence intervals. Therefore, their clinical relevance may be debatable.

**Conclusion:**

OA is associated with lower functional performance, particularly grip strength, compared to those with no disease. These findings highlight the need for exercise-based interventions for individuals with OA, as guidelines suggest.

## 1 Introduction

Chronic lifestyle-related diseases such as OA, diabetes, and HT are highly prevalent worldwide and represent a major public health burden. In Germany, approximately 17.9% of adults report having OA [1,2].5% are diagnosed with diabetes [3], and over 30% live with HT [4], particularly in adults. These conditions not only diminish quality of life and daily functioning but also contribute significantly to rising healthcare costs and long-term disability [5].

To reduce this burden, clinical trials and public health initiatives have increasingly focused on non-pharmacological interventions. Evidence-based clinical guidelines consistently recommend exercise as a first-line treatment for managing OA, diabetes, and HT [[6], [7], [8]]. Regular physical activity is associated with improvements in strength, mobility, blood glucose regulation, and cardiovascular health, ultimately aiming to restore function and mitigate the impact of disease [9].

Despite these recommendations, studies have shown that exercise is under-prescribed in routine clinical practice, especially for patients with OA. Compared to patients with diabetes or HT, individuals with OA are less likely to receive exercise advice from their general practitioner [2,10]. The reasons for this gap in care remain unclear but may include clinician perceptions of patient functioning, time constraints, or patient-level beliefs and health literacy.

Exercise therapy is recommended for all three conditions—OA, diabetes, and HT—but it is unclear whether patients across these diseases have comparable levels of functional limitation and therefore the same degree of clinical need to improve. If functional performance differs substantially between disease groups, this may help explain why exercise advice is not provided equally in routine clinical practice, particularly for individuals with OA [10,11]. Evaluating objective functional performance across these disease groups can therefore offer important context. Although our study does not directly assess physician decision-making, differences in functional performance may help explain why individuals with OA receive exercise advice less frequently than patients with diabetes or HT [10]. Accordingly, we examine whether individuals with OA—alone or in combination with diabetes and/or HT—differ in functional performance compared with those with diabetes, HT, or no disease, using objective measures of grip strength, chair rise time, and walking speed.

## 2 Methods

### 2.1 Source of data and participants

Data for this study were obtained from the Survey of Health, Ageing, and Retirement in Europe (SHARE), a multinational research initiative designed to longitudinally explore the challenges and opportunities associated with the aging process [12,13]. The study began in 2004 with Wave 1, encompassing 12 countries. Since then, data have been collected approximately every two years, with the number of participating countries gradually expanding. As of 2023, SHARE includes 29 countries and covers data from eight main waves of data collection. For the current analysis, we utilized data from Wave 2 (2006), the only wave that includes complete information on all outcomes relevant to this study. The SHARE Wave 2 dataset includes responses from both new and returning participants aged 50 years and older. However, the dataset also includes a small number of younger individuals, such as spouses or partners of eligible participants. To align with our study's focus on older adults, we excluded all individuals under the age of 50 from the analysis. It features a core questionnaire consisting of 22 modules, as well as a dedicated ‘End of Life’ interview conducted with proxies for deceased participants. All data were collected through face-to-face, computer-aided personal interviews, supplemented by self-completion paper and pencil questionnaires.

In addition to the original participating countries (Austria, Germany, Sweden, Netherlands, Spain, Italy, France, Denmark, Greece, Switzerland, Belgium, and Israel), two new EU member states, the Czech Republic and Poland, as well as Ireland, joined the initiative during the second SHARE wave.

### 2.2 Outcome variables

Our dependent variables focused on three available distinct functional performance tests: the maximal grip strength test, the chair rise test, and a walking speed test. Grip strength was measured in kilograms using a Smedley S Dynamometer (TTM, Tokyo; 100 kg capacity). Participants were instructed to grasp the handle as forcefully as possible, with two alternating measurements taken from both the left and right hands. Following the methodology recommended by the SHARE developers [14], we used the maximum recorded value across all attempts in our analysis. For the chair rise test, participants were asked to stand up from a chair and sit back down five times, with the total time required to complete the task (in seconds) recorded by the interviewer. This test was administered only to participants under the age of 75. Participants aged 75 years or older instead completed a walking speed test, in which they walked a 2.5-m distance at their usual pace. The interviewer recorded the time in seconds (to two decimal places) for two walking trials, and the average of these two times was calculated and used as the walking speed outcome.

### 2.3 Independent variables

Our independent variables of interest focused on the presence of three chronic conditions: diabetes, HT, and symptomatic OA, as well as combinations thereof. Participants were asked whether a medical doctor had ever diagnosed them with diabetes or HT, with responses recorded as yes or no. For the identification of symptomatic OA, a stricter criterion was applied: participants had to report both a medical diagnosis of OA and the presence of joint pain within the past six months. Only those who met both conditions were classified as having symptomatic OA; all others were coded as not having the condition. Based on these definitions, we created eight distinct groups reflecting all possible combinations of the three diseases: No disease, Only diabetes, Only HT, Only OA, diabetes and HT, diabetes and OA, HT and OA, and all three diseases. Participants who reported none of the three chronic conditions were defined as a control group.

### 2.4 Confounders

We included the following variables as potential confounders: body mass index (BMI), sex, age, and educational level. BMI was calculated using the participant's self-reported weight and height. Sex was self-reported as either male or female. Age was calculated by subtracting the participant's year of birth from 2007, corresponding to the year of SHARE Wave 2 data collection. Educational level was categorized based on the International Standard Classification of Education (ISCED-97) codes provided in the dataset. Details of the mapping and grouping are provided in the supplementary material. In supplementary analyses, we further considered the covariates—physical activity level, number of comorbidities, and country (as fixed effects)—to assess the robustness of our findings.

### 2.5 Statistical methods

Before conducting the analyses, we applied a series of data preprocessing steps to ensure data quality and reliability.

We began by removing individuals younger than 50 years, in line with our inclusion criterion of focusing on adults aged 50 years and older. Then, we selected participants who reported valid values for our key variables. We excluded participants with missing data on our independent variables OA, diabetes, and HT. Individuals with implausible body mass values—specifically, a weight below 10 kg or height under 100 cm—were excluded to remove biologically unrealistic or erroneous entries. Participants with missing data on the confounders —including age, BMI, female, educational level, knee pain, physical activity, country, and number of diseases— were excluded. Outcome-based exclusions (missing grip strength, chair stand, or walking speed) were applied after age stratification, because different tests were administered in participants <75 and ≥ 75 years.

To account for differences in test administration based on age, we created two distinct analytical datasets:

The MaxChair dataset included participants younger than 75 years who had completed both the grip strength and chair rise tests.

The MaxWalk dataset included participants aged 75 years and older who had completed both the grip strength and walking speed tests.

All the following steps were performed separately for both datasets.

Participants were classified into eight mutually exclusive groups based on self-reported diagnoses of symptomatic OA, HT, and diabetes. Thus, these groups included individuals with no, a single (e.g., Only OA, Only HT, or Only diabetes), those with combinations of two conditions, and finally those with all three conditions. To adjust for confounding factors, we conducted ordinary least squares regression analyses for each outcome in the seven patient groups. The independent variables of interest in the models were the seven disease group indicators, with the category of No disease serving as the reference category. All models were adjusted for age, sex, BMI, and educational level. We assessed model assumptions—such as linearity, homoscedasticity, and normality of residuals—using diagnostic plots. Results were reported as regression coefficients with 95% confidence intervals and were visualized using coefficient plots to support interpretation across outcomes and disease combinations.

All data analyses were performed in Python version 3.11 using Scikit-learn, Pandas, and NumPy libraries. Additional statistical testing and visualizations were conducted using Seaborn, Matplotlib, Statsmodels, and SciPy.

### 2.6 Supplementary analysis

To assess potential selection bias due to the exclusion of participants with missing or implausible data, we compared key demographic and disease characteristics between included and excluded participants. We also re-estimated the regression models, including physical activity, number of comorbidities, and country as additional potential confounding variables.

## 3 Results

### 3.1 Sample characteristics

**Fig. 1:**
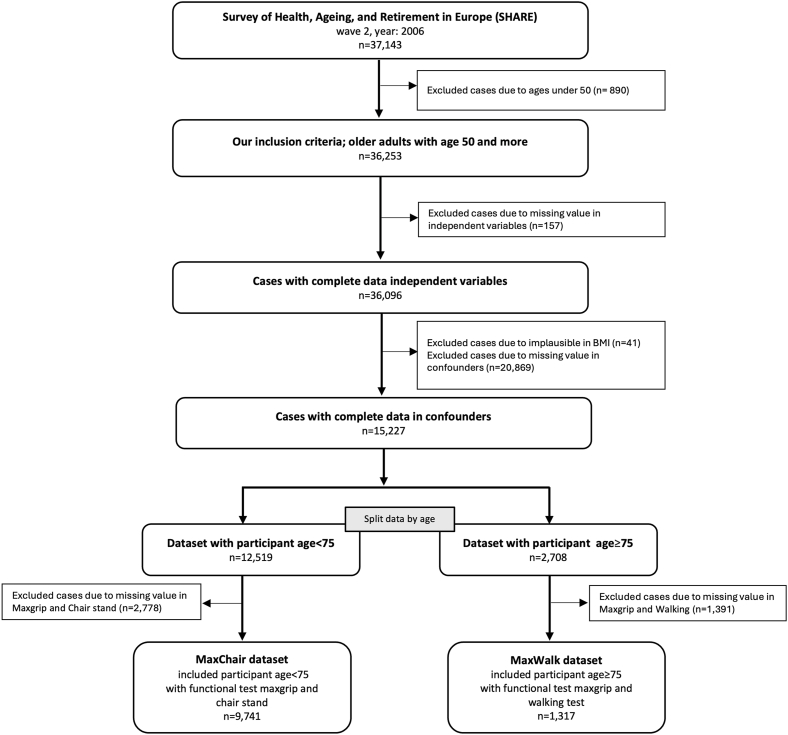
Flowchart of patient selection and data exclusion criteria for the study, showing initial cohort size and subsequent exclusions due to specific criteria and missing data.

A total of 37,143 participants were included in the raw dataset. We first removed 890 individuals younger than 50 years to align with the study's focus on older adults. Next, we excluded 157 participants with missing data in our independent variables (symptomatic OA, HT, or diabetes) (Fig. 1)

**Fig. 2:**
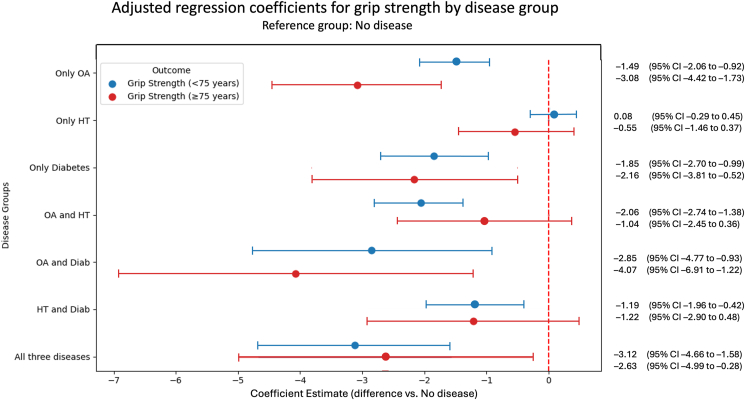
Adjusted regression coefficients for grip strength by disease group in adults aged <75 years (blue) and ≥75 years (red). Each point represents the estimated mean difference in grip strength (kg) compared to the reference group (No disease), based on OLS regression models adjusted for age, BMI, sex, and educational level. Horizontal lines represent 95% confidence intervals. Negative values indicate lower grip strength relative to the reference group. The red dashed vertical line marks the null value (no difference).

After exclusions due to missing values, we excluded individuals with implausible anthropometric values, removing 30 participants with a weight ≤10 kg and 11 participants with a height ≤100 cm. In addition, 474 participants were excluded due to missing weight, and 20,257 due to missing height. After these exclusions, 15,324 participants remained with plausible and complete weight and height information, which are required to calculate BMI. Next, we excluded 97 participants with missing data in one or more of the remaining confounders (age, BMI, sex, educational level, knee pain, physical activity, country, and number of diseases). This resulted in a sample of 15,227 participants aged 50 years or older with complete confounder and independent variables (Fig. 1).

Because different functional tests were administered depending on age, the dataset was then split at age 75. Among the 12,519 participants younger than 75 years, 2778 were excluded due to missing grip strength or chair stand measurements, resulting in a final MaxChair dataset of 9741 participants. Among the 2708 participants aged 75 years or older, 1391 were excluded due to missing grip strength or walking speed values, leaving a final MaxWalk dataset of 1317 participants. Descriptive characteristics for both final datasets are shown in Table 1, Table 2.

**Table 1:** Characteristics of participants under 75 years of age. The total number of cases in this dataset is 9753.

	Variable	No disease	Only OA	Only HT	Only Diabetes	OA and Diab	OA and HT	HT and Diab	All three diseases
0	**Number of cases(**%)	5607 (57.56)	704 (7.23)	2127 (21.84)	290 (2.98)	55 (0.56)	491 (5.04)	379 (3.89)	88 (0.90)
1	**Age; mean (SD)**	58.9 ± 6.5	60.2 ± 6.6	61.5 ± 6.6	62.2 ± 6.5	61.5 ± 6.7	62.9 ± 6.6	63.3 ± 6.3	63.5 ± 6.9
2	**Female; n, (**%**)**	2886 (51.5)	468 (66.5)	1081 (50.8)	106 (36.6)	33 (60.0)	3160 (64.4)	1800 (47.5)	580 (65.9)
3	**BMI; mean (SD)**	25.9 ± 4.0	26.2 ± 4.0	27.9 ± 5.3	27.9 ± 4.5	28.9 ± 5.0	28.4 ± 4.5	29.8 ± 4.7	32.3 ± 5.1
4	**Educational level**								
	-Low (%)	1890 (33.7)	293 (41.6)	798 (37.5)	129 (44.5)	30 (54.5)	226 (46.0)	154 (40.6)	41 (46.6)
	-Medium (%)	2237 (39.9)	258 (36.6)	830 (39.0)	103 (35.5)	14 (25.5)	180 (36.7)	150 (39.6)	28 (31.8)
	-High (%)	1374 (24.5)	124 (17.6)	453 (21.3)	48 (16.6)	10 (18.2)	69 (14.1)	65 (17.2)	12 (13.6)
	-Other (%)	106 (1.9)	29 (4.1)	46 (2.2)	10 (3.4)	1 (1.8)	16 (3.3)	10 (2.6)	7 (8.0)
5	**Grip strength (kg); mean (SD)**	38.2 ± 11.5	33.6 ± 11.6	37.7 ± 11.7	38.0 ± 11.0	33.2 ± 11.8	32.7 ± 11.2	36.6 ± 11.5	31.7 ± 11.5
6	**Chairs stand (sec); mean (SD)**	10.9 ± 7.2	12.1 ± 6.5	11.1 ± 6.0	10.9 ± 5.4	13.3 ± 6.8	120 ± 5.0	12.7 ± 9.4	12.7 ± 5.5

**Table 2:** The characteristics of participants aged 75 and over. The dataset contains 1331 cases.

	Variable	No disease	Only OA	Only HT	Only Diabetes	OA and Diab	OA and HT	HT and Diab	All three diseases
0	**Number of cases(**%)	501 (38.04)	123 (9.34)	375 (28.47)	76 (5.77)	23 (1.75)	112 (8.50)	72 (5.47)	35 (2.66)
1	**Age; mean (SD)**	80.1 ± 3.8	80.6 ± 3.8	80.3 ± 4	79.8 ± 3.3	78.7 ± 3.1	80.4 ± 4	80.3 ± 3.3	79.6 ± 3.5
2	**Female; n, (**%**)**	222 (44.3)	73 (59.3)	210 (56)	37 (48.7)	9 (39.1)	65 (58)	44 (61.1)	22 (62.9)
3	**BMI; mean (SD)**	25.3 ± 4.4	26.5 ± 6.4	26.1 ± 3.7	26.9 ± 3.8	27.4 ± 5.5	27.3 ± 3.8	27.4 ± 4.1	29.1 ± 4.1
4	**Educational level**								
	-Low (%)	253 (50.5)	76 (61.8)	204 (54.4)	47 (61.8)	13 (56.5)	60 (53.6)	34 (47.2)	13 (37.1)
	-Medium (%)	140 (27.9)	16 (13)	98 (26.1)	13 (17.1)	7 (30.4)	26 (23.2)	19 (26.4)	11 (31.4)
	-High (%)	78 (15.6)	22 (17.9)	52 (13.9)	8 (10.5)	0	17 (15.2)	14 (19.4)	5 (14.3)
	-Other (%)	30 (6)	9 (7.3)	21 (5.6)	8 (10.5)	3 (13)	9 (8)	5 (6.9)	6 (17.1)
5	**Grip strength (kg); mean (SD)**	29.4 ± 9.3	24.3 ± 9.1	27.4 ± 9.4	27 ± 8.7	26.7 ± 6.9	26.7 ± 10.8	26.2 ± 8	24.9 ± 8.7
6	**Walking speed (sec); mean (SD)**	4.7 ± 3.7	5.3 ± 3.8	4.8 ± 3.6	5.5 ± 4.7	5.5 ± 3.2	5.2 ± 3.9	5.7 ± 4.7	5.3 ± 2.9

### 3.2 Functional performance in participants aged under 75 years

Descriptive statistics showed that individuals under 75 years with Only OA had lower mean grip strength (33.6 ± 11.6 kg) compared to those with Only HT (37.7 ± 11.7 kg) or Only diabetes (38.0 ± 11 kg) (Table 1). Time spent for the chair stand test was also longer (indicating worse lower-limb function) in the Only OA group (12.1 ± 6.5 s) compared to the Only HT (11.1 ± 6.0 s) and the Only diabetes groups (10.9 ± 5.4 s).

In addition, we observed that grip strength and chair stand performance declined with more chronic conditions. Grip strength dropped from 38.2 kg (No disease) to 37.7 kg (Only HT), 36.6 kg (HT and Diabetes), 33.2 kg (OA and Diabetes), 32.7 kg (OA and HT), and 31.7 kg (All three diseases). Chair stand time increased from 10.9 s to 11.1 s (Only HT), 10.9 s (Only diabetes), 13.3 s (OA and Diabetes), 12.7 s (HT and Diabetes), and 12.7 s (All three diseases), showing reduced functional performance with higher multimorbidity.

### 3.3 Functional performance in adults aged 75 years and older

Descriptive statistics showed that individuals aged 75 and above, patients with Only OA had the lowest mean grip strength (24.3 ± 9.1 kg) compared to those with Only HT (27.4 ± 9.4 kg) and Only diabetes (27 ± 8.7 kg) (Table 2). Walking speed was also slowest in the Only OA group (5.3 ± 3.8 s), compared to Only HT (4.8 ± 3.6 s) and Only diabetes (5.5 ± 4.7 s).

In addition, we observed that both grip strength and walking speed worsened with more chronic conditions. Grip strength fell from 29.4 kg (No disease) to 26.7 kg (OA and Diabetes), 26.7 kg (OA and HT), 26.2 kg (HT and Diabetes), and 24.9 kg (All three diseases). Walking time increased from 4.7 s to 5.5, 5.2, 5.7, and 5.3 s, respectively, reflecting a steady decline in functional performance with higher disease burden.

#### 3.3.1 Regression analysis for maxgrip in both age groups

Among participants younger than 75 years, individuals with Only OA showed lower grip strength compared with the No disease group (−1.49 kg; 95% CI: −2.06 to −0.92). A similar pattern was observed for the Only diabetes group (−1.85 kg; 95% CI: −2.70 to −0.99). For individuals with Only HT, No difference was observed compared to those with NO diseases (0.08 kg; 95% CI: −0.29 to 0.45). Participants with combinations involving OA—such as OA and HT (−2.06 kg; 95% CI: −2.74 to −1.38), OA and Diabetes (−2.85 kg; 95% CI: −4.77 to −0.93), or all three diseases (−3.12 kg; 95% CI: −4.66 to −1.58)—demonstrated larger point estimate differences in grip strength relative to the No disease group.

Among participants aged 75 years and older, individuals with Only OA also showed lower grip strength compared with those in the No disease group (−3.08 kg; 95% CI: −4.42 to −1.73). Individuals with Only diabetes similarly showed lower grip strength (−2.16 kg; 95% CI: −3.81 to −0.52). For individuals with Only HT, no difference was found compared to those with No disease (−0.55 kg; 95% CI: −1.46 to 0.37). Differences were also observed in groups with OA and diabetes (−4.07 kg; 95% CI: −6.91 to −1.22) and all three diseases (−2.63 kg; 95% CI: −4.99 to −0.28) (Fig. 2).

Observing the CIs, there is no overlap for the patient group with OA only and diabetes only to the HT only patient group in the grip strength test, which could indicate a difference between patient groups.

#### 3.3.2 Regression analysis for chair stand and walking speed in both age groups

Among participants younger than 75 years, individuals with Only OA showed longer chair stand times compared with No disease group (0.83 s; 95% CI: 0.29 to 1.37). Individuals with HT and diabetes also showed longer chair stand times compared with No disease group (1.15 s; 95% CI: 0.42 to 1.87). For the remaining patient groups, estimated differences were smaller, and confidence intervals included zero, indicating no evidence of a difference.

Observing the CIs, there is no overlap for the patient group with OA only and HT only in the chair stand test, which could indicate a difference between patient groups.

**Fig. 3:**
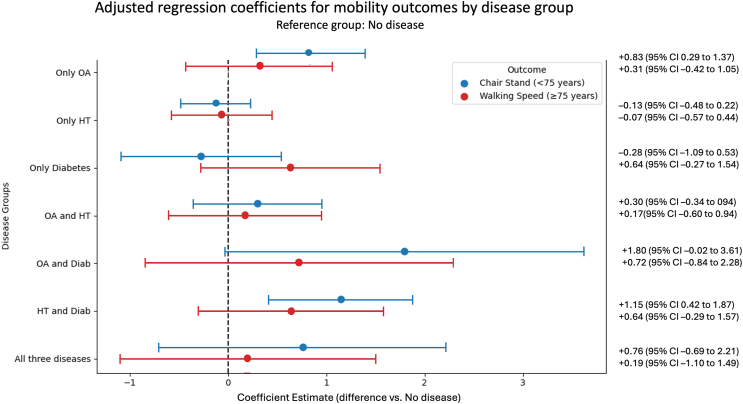
Adjusted regression coefficients for mobility outcomes by disease group in adults aged <75 years (blue: chair stand time) and ≥75 years (red: walking speed). Each point represents the estimated mean difference in performance compared to the reference group (No disease), based on OLS regression models adjusted for age, BMI, sex, and educational level. Horizontal lines indicate 95% confidence intervals. For both mobility outcomes, negative values indicate worse performance relative to the no-disease group (i.e., longer chair stand time or slower walking speed). The dashed vertical line at zero indicates no difference.

In participants aged 75 years and older, estimated differences in walking speed for all disease groups had confidence intervals that included zero. (Fig. 3).

### 3.4 Supplementary analysis

In addition to the primary models, we adjusted for physical activity, comorbidities, and country to produce effect estimates that were highly consistent with the primary findings (Table S1).

Among the 37,143 participants initially considered, 21,772 were excluded primarily due to missing or implausible data on key variables. Comparisons between included and excluded groups showed a difference in mean age of two years (64 vs. 66 years). However, no substantial differences were observed for sex distribution, BMI, or the prevalence of OA, HT, or diabetes (Table S1), suggesting minimal risk of selection bias introduced by these exclusions. Standardized mean differences ranged from 0.016 to 0.283, indicating generally modest imbalance between groups.

## 4 Discussion

### 4.1 Key findings

This study examined whether individuals with OA demonstrate different levels of functional performance than those with diabetes, HT, or No disease. Using a representative sample from SHARE Wave 2 and objective functional performance tests, we found in our descriptive statistics that participants with OA have lower grip strength than those with HT and diabetes only. In the lower leg function, they performed worse than patients with HT and similar to those with diabetes.

Our adjusted analyses showed differences in the grip strength test between all patient groups, but HT only, and the no disease group among patients younger than 75 years. A similar trend was observed for those at least 75 years old, though here no statistical significance difference was reached by three patient groups: HT only, OA and HT, and HT and diabetes. For both age groups, the differences in point estimates between patient groups were small with overlapping confidence intervals. An expectation could only be seen between patients with only OA, only diabetes, and only HT for the grip strength test, where the CIs did not overlap, which could indicate that patients with HT have less grip strength compared to the other two patient groups.

Fewer differences were observed for the lower leg functional tests. Statistically significant differences were only found for two patients younger than 75years: OA-only patients, and patients with HT and diabetes managed fewer chair stand tests than those with no diseases. Again, the CIs overlapped for all patient groups but among those younger than 75 years with OA only and HT only, which could indicate a difference between these patient groups.

### 4.2 Interpretation of results

These findings challenge the perception that OA patients maintain better function than those with other chronic conditions. In our descriptive findings, participants with OA tended to show slightly lower functional performance—particularly in grip strength and chair rise—compared with those with diabetes or HT. However, in adjusted analyses, the magnitude of these differences was small and did not reach established thresholds for clinical relevance, indicating broadly comparable levels of functional performance across disease groups. Though previous research shows that patients with OA are least likely to receive respected advice from the general practitioners [10]. Given the observational nature of our study, these findings should be interpreted as associations rather than causal relationships.

Grip strength was the most sensitive and consistent marker, aligning with previous research showing its relevance as a global indicator of aging and physical well-being [15,16].

In adjusted analyses, participants with OA and those with diabetes tended to show lower grip strength compared with the No disease group. Participants with HT showed no difference. Across outcomes, estimated differences were generally small in magnitude and did not reach established thresholds for clinical relevance.

Chair rise time tended to be slightly longer among participants with OA, indicating modest differences in lower-body performance. Since the knee is one of the most commonly affected joints in OA [17], this highlights the need to consider OA location when interpreting such tests in clinical assessments.

Among participants aged 75 years and older, differences in walking speed between disease groups were generally inconclusive, with confidence intervals frequently including zero. Since this was a secondary data analysis, no formal power calculation was conducted. Nonetheless, to evaluate the clinical relevance of our findings, we compared the adjusted deficits associated with OA to established Minimal Clinically Important Difference thresholds. The Minimal Clinically Important Difference for meaningful functional decline in grip strength is typically 5–6.5 kg [18]. A clinically meaningful change in chair-stand performance is approximately 2.6–3 s [19], and for walking speed, 0.10 m/s is considered clinically meaningful [20]. Thus, although our disease groups differed statistically, the magnitude of these differences did not reach established thresholds for clinical relevance. This underpins that, despite statistically detectable variation, all patient groups—including those with OA, HT, or diabetes—should be advised to engage in physiotherapy and exercise-based interventions to the same extent, as recommended by current clinical guidelines [[21], [22], [23]].

### 4.3 Strengths and limitations

A major strength of this study is its use of a large, diverse sample from multiple European countries, enhancing generalizability. Functional outcomes were assessed using standardized, objective tests, minimizing measurement bias [[24], [25], [26]].

However, some limitations should be considered. Several key variables, including disease status (OA, diabetes, and HT), body weight and height, and selected covariates, were based on self-report, which may introduce recall bias or misclassification. The "no disease" group may still include individuals with other health issues, though our disease groups may also suffer from these diseases.

Additionally, 21,772 participants were excluded due to missing or implausible anthropometric data—specifically, 30 individuals with weight ≤10 kg, 11 with height ≤100 cm, 474 with missing weight, and 20,257 with missing height—which prevented valid BMI calculation, which may have introduced selection bias. These implausible values produced extreme BMI estimates and were therefore removed during preprocessing.

Although differences between included and excluded participants were modest for most variables, standardized mean differences ranged up to 0.283, exceeding commonly used thresholds (e.g., SMD >0.1) for meaningful imbalance. This suggests that excluded individuals may have had higher BMI and overall poorer health, resulting in a potentially healthier analytic sample. Nevertheless, additional analyses adjusting for physical activity, comorbidities, and country produced estimates consistent with the primary findings (Table S1).

Second, we relied solely on SHARE Wave 2 data from 2006, as it is the only wave that includes complete information on all three objective functional performance tests (grip strength, chair rise, and walking speed). Importantly, SHARE is a longitudinal panel designed to repeatedly sample the 50+ European population, and demographic and health characteristics have remained remarkably stable across waves. Published analyses using later waves (e.g., easySHARE Release 8.0.0) show consistent age and sex structures, similar distributions of BMI, and only modest changes in the prevalence of chronic conditions such as HT, diabetes, and OA in comparison to the characteristics of our sample [27,28]. Functional measures available in later waves—particularly grip strength in Waves 5 and 6—also demonstrate comparable age-specific averages, reflecting gradual age-related decline rather than methodological or time dependent differences. These findings suggest that characteristics of participants in Wave 2 closely resemble those in more recent waves, supporting the generalizability of our descriptive results. In summary, while this dataset is relatively old, there is no strong evidence to suggest that population-level associations between chronic disease burden and functional performance have changed dramatically in the intervening years [29]. Age-related declines in muscle strength and performance remain well-documented and are biologically driven processes. Because our analysis is descriptive rather than predictive, internal validation procedures such as split-sample or bootstrap approaches were not applicable. Nevertheless, relying on a single wave limits generalizability, and confirming these findings in more recent or independent datasets would strengthen robustness. SHARE Wave 2 provides only three objective performance tests (grip strength, chair rise, walking speed), so joint-specific OA limitations or balance/mobility tasks could not be assessed, but would be of interest as well.

Finally, because this study looks at data from only one point in time, we can't say for sure whether chronic diseases cause changes in functional performance. As this study is based on cross-sectional data, we cannot determine whether chronic diseases cause reduced functional performance or otherwise.

### 4.4 Implications

Our findings indicate that individuals with OA, including those with comorbidities, demonstrate levels of functional performance that are broadly comparable to those observed in individuals with other chronic conditions. Although our study does not assess the reasons behind differences in exercise advice as shown in previous studies [10], the small observed functional differences in functional performance suggest that patients with OA have a similar need for exercise-based interventions as patients with other chronic conditions. Recognizing these impairments may help contextualize reported differences in exercise advice across conditions, but cannot explain them.

## 5 Conclusion

This study provides evidence that individuals with OA, including those with additional chronic conditions, demonstrate broadly similar levels of functional performance compared to those with diabetes and HT alone or in combination. The small differences in functional performance observed between these disease groups may not be readily apparent in routine care assessments. Recognizing the functional impairments associated with OA is essential to ensure that these patients receive appropriate, evidence-based exercise recommendations.

## Availability of data

The datasets analyzed during the current study are distributed by SHARE-ERIC to registered users through the SHARE Research Data Center. (Börsch-Supan 2022)

Data from easySHARE release 8.0.0 (https://doi.org/10.6103/SHARE.easy.800), SHARE Wave 2 Release 8.0.0 (https://doi.org/10.6103/SHARE.w8.800)

## Author contributions

Mahdie Rafiei: Conceptualization, Methodology, Formal Analysis, Writing- Original Draft, Writing – Review & Editing.

Niklas Probul: Conceptualization, Methodology, Writing – Review & Editing.

Jan Baumbach: Conceptualization, Methodology, Writing – Review & Editing.

Linda Baumbach: Conceptualization, Methodology, Writing- Original Draft, Writing – Review & Editing.

## Funding

This study was developed as part of the PhysioAI project and is funded by the 10.13039/501100002347Federal Ministry of Research, Technology and Space of Germany(BMFTR) and by the NextGenerationEU Fund of the 10.13039/501100000780European Union under grant number 16DKWN115A.

## Declaration of interest statement

The authors declare no competing financial or personal interests that could have influenced the work reported in this manuscript.

## Abbreviation

OA: osteoarthritis
HT: Hypertension
