# Supplementary material for: Older adults with osteoarthritis show lower functional performance compared to those with diabetes or hypertension: Evidence from the SHARE dataset
Source: Osteoarthr Cartil Open. 2026 Mar 21;8(2):100781. doi: 10.1016/j.ocarto.2026.100781 (PMC13068654; doi:10.1016/j.ocarto.2026.100781)
Supplement: Multimedia component 1 [file mmc1.docx]

# Supplementary Analysis:

## 1. Comparison of included and excluded participants

A total of 37,143 participants were initially available in the SHARE Wave 2 dataset. After applying the predefined inclusion criteria and requiring complete and plausible values for the key variables used in the analyses—age, sex, educational level, physical activity, comorbidities, country, grip strength, chair stand time, and walking speed—a total of 15,227 participants remained in the final analytical sample. The remaining 21,916 participants were classified as excluded cases. Because height and weight information contained numerous missing or implausible values among excluded individuals, we performed an additional data-cleaning step to obtain interpretable descriptive statistics for anthropometric variables.

Height values ≤100 cm and weight values ≤10 kg was considered implausible and recoded as missing. Body mass index (BMI) was calculated only for participants with valid height and weight measurements using the standard formula (kg/m²). This procedure prevented extreme BMI outliers that would result from erroneous entries and ensured that only physiologically meaningful BMI values were used when comparing included and excluded participants. Functional performance measures (grip strength, chair stand time, walking speed) were compared descriptively using all available non-missing values. These steps allowed a systematic comparison of included versus excluded individuals and helped assess potential selection patterns.

|  | **Variable** | **Included (n = 15,227)** | **Excluded (n = 21,916)** |
| --- | --- | --- | --- |
| 1 | **Age, mean (SD)** | 64.1 (9.9) | 66 (10.6) |
| 2 | **BMI, mean (SD)** | 26.8 (4.7) | 25.6 (4.8) |
| 3 | **Female (%)** | 8,195 (53.8%) | 12,550 (57.3%) |
| 4 | **Education (%)** |  |  |
|  | - Low | 6,370 (41.8%) | 9,577 (43.7%) |
|  | - Medium | 5,404 (35.5%) | 6,372 (29.1%) |
|  | - High | 2,890 (19%) | 4,138 (18.9%) |
|  | - Other | 563 (3.7%) | 1,352 (6.2%) |
| 5 | **Physical activity (%)** |  |  |
|  | - More than once a week | 10,667 (70.1%) | 15,533 (70.9%) |
|  | - Once a week | 1,871 (12.3%) | 2,508 (11.4%) |
|  | - One to three times a month | 842 (5.5%) | 970 (4.4%) |
|  | - Never/rarely | 1,847 (12.1%) | 2,522 (11.5%) |
| 6 | **Comorbidities (%)** |  |  |
|  | 0: | 4,270 (28.1%) | 6,720 (30.7%) |
|  | 1: | 4,501 (29.6%) | 6,701 (30.6%) |
|  | 2: | 3,048 (20%) | 4,262 (19.4%) |
|  | 3: | 1,785 (11.7%) | 2,294 (10.5%) |
|  | 4: | 872 (5.7%) | 1,148 (5.2%) |
|  | 5: | 410 (2.7%) | 461 (2.1%) |
|  | 6: | 182 (1.2%) | 181 (0.8%) |
|  | 7: | 96 (0.6%) | 93 (0.4%) |
|  | 8: | 34 (0.2%) | 36 (0.2%) |
|  | 9: | 14 (0.1%) | 12 (0.1%) |
|  | 10: | 10 (0.1%) | 4 (0%) |
|  | 11: | 3 (0%) | 2 (0%) |
|  | 12: | 1 (0%) | 1 (0%) |
|  | 13: | 1 (0%) | 1 (0%) |
| 7 | **Country (%)** |  |  |
|  | Country: Austria | 72 (0.5%) | 1,122 (5.1%) |
|  | Country: Belgium | 338 (2.2%) | 2,889 (13.2%) |
|  | Country: Czech Republic | 2,595 (17%) | 140 (0.6%) |
|  | Country: Denmark | 1,279 (8.4%) | 1,351 (6.2%) |
|  | Country: France | 915 (6%) | 2,075 (9.5%) |
|  | Country: Germany | 982 (6.5%) | 1,646 (7.5%) |
|  | Country: Greece | 851 (5.6%) | 2,559 (11.7%) |
|  | Country: Ireland | 984 (6.5%) | 51 (0.2%) |
|  | Country: Israel | 556 (3.7%) | 1,892 (8.6%) |
|  | Country: Italy | 1,167 (7.7%) | 1,813 (8.3%) |
|  | Country: Netherlands | 854 (5.6%) | 1,829 (8.3%) |
|  | Country: Poland | 2,379 (15.6%) | 87 (0.4%) |
|  | Country: Spain | 811 (5.3%) | 1,612 (7.4%) |
|  | Country: Sweden | 713 (4.7%) | 2,083 (9.5%) |
|  | Country: Switzerland | 731 (4.8%) | 767 (3.5%) |
| 8 | **Grip strength (kg), mean (SD)** | 35 (12.1) | 33.6 (11.8) |
| 9 | **Chair stand time (sec), mean (SD)** | 11.23 (6.99) | 11.52 (7.14) |
| 10 | **Walking speed (m/s), mean (SD)** | 5.07 (3.95) | 5.01 (3.91) |

Table S1. Characteristics of included and excluded participants

## 2. Regression models with new confounders

| **Disease group** | **Coefficient (Adj. Mean Diff)** | **95% CI Lower** | **95% CI Upper** | **p-value** |
| --- | --- | --- | --- | --- |
| Only OA | -0.976 | -1.531 | -0.421 | 0.0006 |
| Only HT | 0.555 | 0.157 | 0.954 | 0.0063 |
| Only Diabetes | -0.951 | -1.794 | -0.108 | 0.0270 |
| HT and Diab | 0.238 | -0.586 | 1.062 | 0.5717 |
| OA and Diab | -1.241 | -3.110 | 0.627 | 0.1929 |
| OA and HT | -0.882 | -1.581 | -0.183 | 0.0134 |
| All three diseases | -1.471 | -3.015 | 0.072 | 0.0617 |

Table S2: Regression analysis for maxgrip in age under 75 (No disease as reference) with new confounders

| **Disease group** | **Coefficient (Adj. Mean Diff)** | **95% CI Lower** | **95% CI Upper** | **p-value** |
| --- | --- | --- | --- | --- |
| Only OA | 0.753 | 0.219 | 1.288 | 0.0057 |
| Only HT | -0.351 | -0.735 | 0.032 | 0.0727 |
| Only Diabetes | -0.719 | -1.531 | 0.092 | 0.0822 |
| HT and Diab | 0.546 | -0.247 | 1.340 | 0.1769 |
| OA and Diab | 1.561 | -0.237 | 3.360 | 0.0888 |
| OA and HT | -0.066 | -0.739 | 0.606 | 0.8465 |
| All three diseases | 0.206 | -1.280 | 1.692 | 0.7856 |

Table S3: Regression analysis for chair stand in age under 75 (No disease as reference) with new confounders

| **Disease group** | **Coefficient (Adj. Mean Diff)** | **95% CI Lower** | **95% CI Upper** | **p-value** |
| --- | --- | --- | --- | --- |
| Only OA | -2.237 | -3.551 | -0.922 | 0.0009 |
| Only HT | 0.172 | -0.760 | 1.104 | 0.7176 |
| Only Diabetes | -1.087 | -2.714 | 0.541 | 0.1905 |
| HT and Diab | 0.015 | -1.726 | 1.756 | 0.9866 |
| OA and Diab | -2.419 | -5.217 | 0.380 | 0.0902 |
| OA and HT | 0.428 | -1.013 | 1.869 | 0.5605 |
| All three diseases | 0.434 | -2.015 | 2.883 | 0.7280 |

Table S4: Regression analysis for maxgrip in age 75 or above 75 (No disease as reference) with new confounders

| **Disease group** | **Coefficient (Adj. Mean Diff)** | **95% CI Lower** | **95% CI Upper** | **p-value** |
| --- | --- | --- | --- | --- |
| Only OA | 0.048 | -0.672 | 0.768 | 0.8962 |
| Only HT | -0.225 | -0.736 | 0.285 | 0.3871 |
| Only Diabetes | 0.278 | -0.614 | 1.170 | 0.5411 |
| HT and Diab | 0.365 | -0.589 | 1.319 | 0.4528 |
| OA and Diab | 0.410 | -1.123 | 1.944 | 0.5997 |
| OA and HT | -0.223 | -1.012 | 0.567 | 0.5801 |
| All three diseases | -0.558 | -1.900 | 0.784 | 0.4150 |

Table S5: Regression analysis for maxgrip in age under 75 (No disease as reference) with new confounders

## 3. Standardized Mean Difference (SMD)

To quantify the magnitude of differences between included and excluded participants, we calculated standardized mean differences (SMDs) for demographic, clinical, and functional variables (Table S2).

|  | **Variable** | **Standardized Mean Difference (SMD)** |
| --- | --- | --- |
| 1 | **Age** | 0.187 |
| 2 | **BMI** | 0.257 |
| 3 | **Female** | 0.069 |
| 4 | **Educational level** | 0.283 |
| 5 | **Grip strenght** | 0.117 |
| 6 | **Chair stand** | 0.040 |
| 7 | **Walking speed** | 0.016 |

Table S6. Standardized mean differences (SMDs) between included and excluded participants for demographic, clinical, and functional variables.

## 4. Education Level Classification

Educational level was categorized based on the International Standard Classification of Education (ISCED-97) codes provided in the dataset. These codes were mapped into broader groups: Low (codes 1 and 2), Medium (codes 3 and 4), and High (codes 5 and 6). Responses such as None, Other, Refusal, still in school, and don’t know were grouped under the category Other.

Low: ISCED-97 code 1 and 2

Medium: ISCED-97 code 3 and 4

High: ISCED-97 code 5 and 6

Other: Includes 'None', 'Other', 'Refusal', 'Still in school', and 'Don't know'
